# High-resolution ex vivo magnetic resonance angiography: a feasibility study on biological and medical tissues

**DOI:** 10.1186/1472-6793-10-3

**Published:** 2010-03-12

**Authors:** Anne S Rasmussen, Henrik Lauridsen, Christoffer Laustsen, Bjarke G Jensen, Steen F Pedersen, Lars Uhrenholt, Lene WT Boel, Niels Uldbjerg, Tobias Wang, Michael Pedersen

**Affiliations:** 1 MR-research Centre, Aarhus University Hospital, Aarhus, Denmark; 2 Department of Biology, Aarhus University, Aarhus, Denmark; 3 Department of Forensic Medicine, Aarhus University, Aarhus, Denmark; 4 Department of Gynaecology and Obstetrics, Aarhus University Hospital, Aarhus, Denmark

## Abstract

**Background:**

In biomedical sciences, ex vivo angiography is a practical mean to elucidate vascular structures three-dimensionally with simultaneous estimation of intravascular volume. The objectives of this study were to develop a magnetic resonance (MR) method for ex vivo angiography and to compare the findings with computed tomography (CT). To demonstrate the usefulness of this method, examples are provided from four different tissues and species: the human placenta, a rice field eel, a porcine heart and a turtle.

**Results:**

The optimal solution for ex vivo MR angiography (MRA) was a compound containing gelatine (0.05 g/mL), the CT contrast agent barium sulphate (0.43 mol/L) and the MR contrast agent gadoteric acid (2.5 mmol/L). It was possible to perform angiography on all specimens. We found that ex vivo MRA could only be performed on fresh tissue because formalin fixation makes the blood vessels permeable to the MR contrast agent.

**Conclusions:**

Ex vivo MRA provides high-resolution images of fresh tissue and delineates fine structures that we were unable to visualise by CT. We found that MRA provided detailed information similar to or better than conventional CTA in its ability to visualize vessel configuration while avoiding interfering signals from adjacent bones. Interestingly, we found that vascular tissue becomes leaky when formalin-fixed, leading to increased permeability and extravascular leakage of MR contrast agent.

## Background

In vivo angiography is frequently employed to produce a structural overview of the intravascular configuration in living tissues [1] whereas angiography of excised organs or post-mortem angiography is rarely performed [2-4]. For example, ex vivo angiography has been used successfully to identify structural components of carotid atherosclerotic plaques [5] and to visualize the renal microvasculature [6]. In addition, post mortem angiography provides information secondary to conventional autopsy in humans and animals [7,8]. Computed tomography angiography (CTA) can be employed with sub-millimetre resolution of vessels in conjunction with an intravascular contrast agent [9]. However, accurate delineation of the entire vessel configuration acquired by CTA is often complicated by the inherent absorption of X-rays in bones and cartilage, characterized as hyperintense areas on CTA images. Magnetic resonance angiography (MRA) is another method, where an intravascular confined paramagnetic compound generates a change in the local magnetization of water, resulting in a hyperintense signal of the streaming blood. MRA has potential advantages over CTA in its unique ability to reveal the intravascular compartment without concomitant contributions from bone and cartilage. On the other hand, the small molecules of most available magnetic resonance contrast agents leak rapidly into the interstitial compartment, hampering detailed and prolonged intravascular measurements using this technique. In clinical situations, this extravasation from the intravascular compartment is overcome by performing an MRA procedure in which images are acquired during the first pass of the agent through the arteries. However, as rapid circulation of blood in smaller species and the lack of circulation in excised organs and dead subjects preclude the use of this approach, there are today no available MRA techniques allowing three-dimensional (3D) high-resolution images of blood vessels in these situations.

This methodological study presents a novel method for ex vivo MRA. To demonstrate the usefulness of this method, examples of both zoological and biomedical relevance were studied from four distinctly different tissues: the human placenta, a rice field eel, a porcine heart and a turtle.

## Results and Discussion

The experimental study was conducted in several steps. Prior to MRA and CTA of selected specimens, we defined the optimal concentration of contrast agents. Next, the use of formalin-fixed and fresh specimens was elucidated to reveal the microstructural consequences of protein binding in the presence of formalin. Three different specimens were then subjected to the chosen MRA and CTA protocols. First, fresh placentas were harvested following elective caesarean section from our obstetric department. Second, live specimens of rice field eel (*Monopterus albus*) were purchased from aquacultures in Vietnam. They were kept in a 500 L plastic aquarium in a 12:12 h L:D routine, kept at 29°C and fed with mussels and shrimps. Third, excised hearts were used from healthy female Danish Landrace pigs weighing 65 kg. Fourth, turtles (*Trachemys scripta*) were examined to demonstrate the proposed MRA and CTA methods on a complete animal.

### Preparation of contrast agent

**Figure 1:**
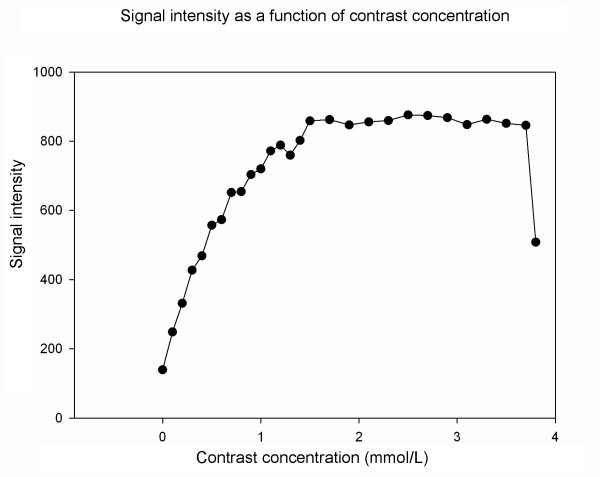
**Optimization of concentration of the contrast agent**. MR scanning of tubes containing contrast agent at different concentrations was performed. The signal intensity was plotted as a function of concentration. The signal intensity increased with contrast concentration up to 2 mmol/L followed by a steady decline so we decided to use 2.5 mmol/L in subsequent MRA studies. Data were acquired using a 3D gradient-echo sequence with the following parameters: thickness 1 mm; TR 23.1 ms; TE 1.6 ms and excitation flip angle 30°.

1. An MRA protocol was applied to image tubes with various gadolinium concentrations (0-3.5 mmol/L), containing the magnetic resonance contrast agent gadoteric acid (Dotarem; Guerbet, France), to determine the concentration that generated the highest image signal. The relationship between concentration and signal is shown in Figure 1 and a concentration of 2.5 mmol/L was considered optimal with the MRA sequence and parameters given in the "Methods" section. Optimization of CT contrast concentration was not performed since development of a contrast solution for MRA was the main purpose of the study.

2. Two different contrast agent solutions were prepared. For contrast mix 1, saline was heated to 60°C and gelatine at a final concentration of 0.05 g/mL (gelatine for microbiology, Merck, Darmstadt, Germany) was added in small portions while stirring together with the CT contrast agent barium sulphate at a final concentration of 0.43 mol/L (Mixobar Colon, 1 g/mL; Astra Tech, Sweden). Dotarem was added to a final concentration of 2.5 mmol/L when the solution had cooled to 40°C. Contrast mix 2 contained saline and the following ingredients: the MR contrast agent gadofosveset trisodium (Vasovist; Bayer-Schering Pharma, Germany) at 2.5 mmol/L and albumin at 60 g/L (egg white albumin; Sigma-Aldrich, USA). The saline was heated to 40°C, and albumin and Vasovist were added while stirring. As Vasovist binds to albumin, the gadolinium-containing compound cannot diffuse from the intra- to the extravascular compartment and Vasovist is therefore considered a suitable intravascular contrast agent for living specimens. Additionally, a commercially made CT contrast agent, Microfil (Flow Tech, USA), was used for CTA of the rice field eel head, which was compared with the acquired MRA [10].

Comparing results obtained with contrast mixes 1 and 2 (data not shown) demonstrated that contrast mix 1 was better by serving both as an MRA and a CTA agent. This property facilitates consecutive MRA and CTA on the same sample. However, contrast mix 2 may be of benefit thanks to its lower viscosity and easier distribution to small capillary vessels.

### Microstructural effect of formalin fixation

**Figure 2:**
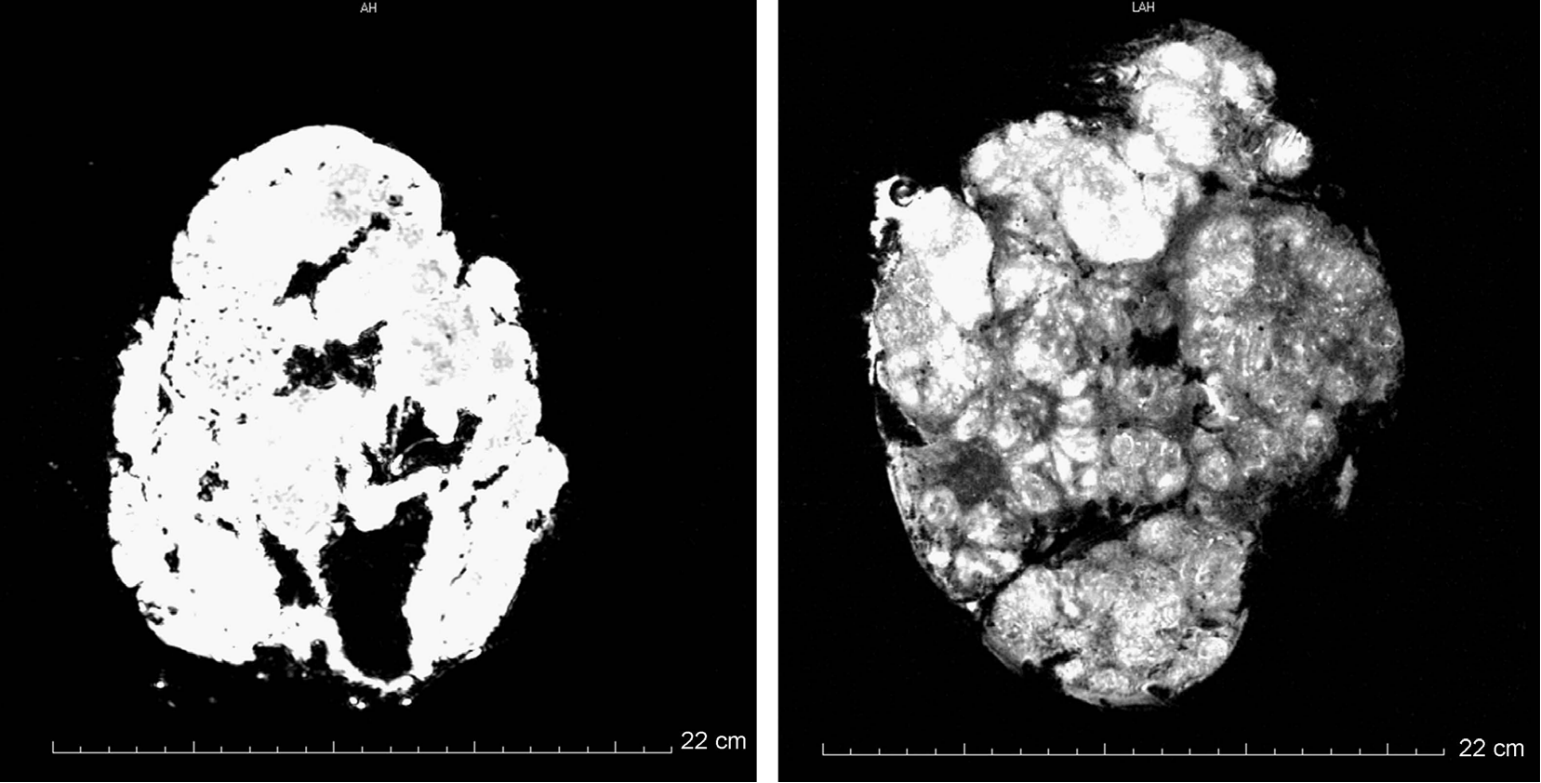
**Left: MRA of formalin-fixed placenta prior to MRA**. Right: MRA of fresh placenta.

**Figure 3:**
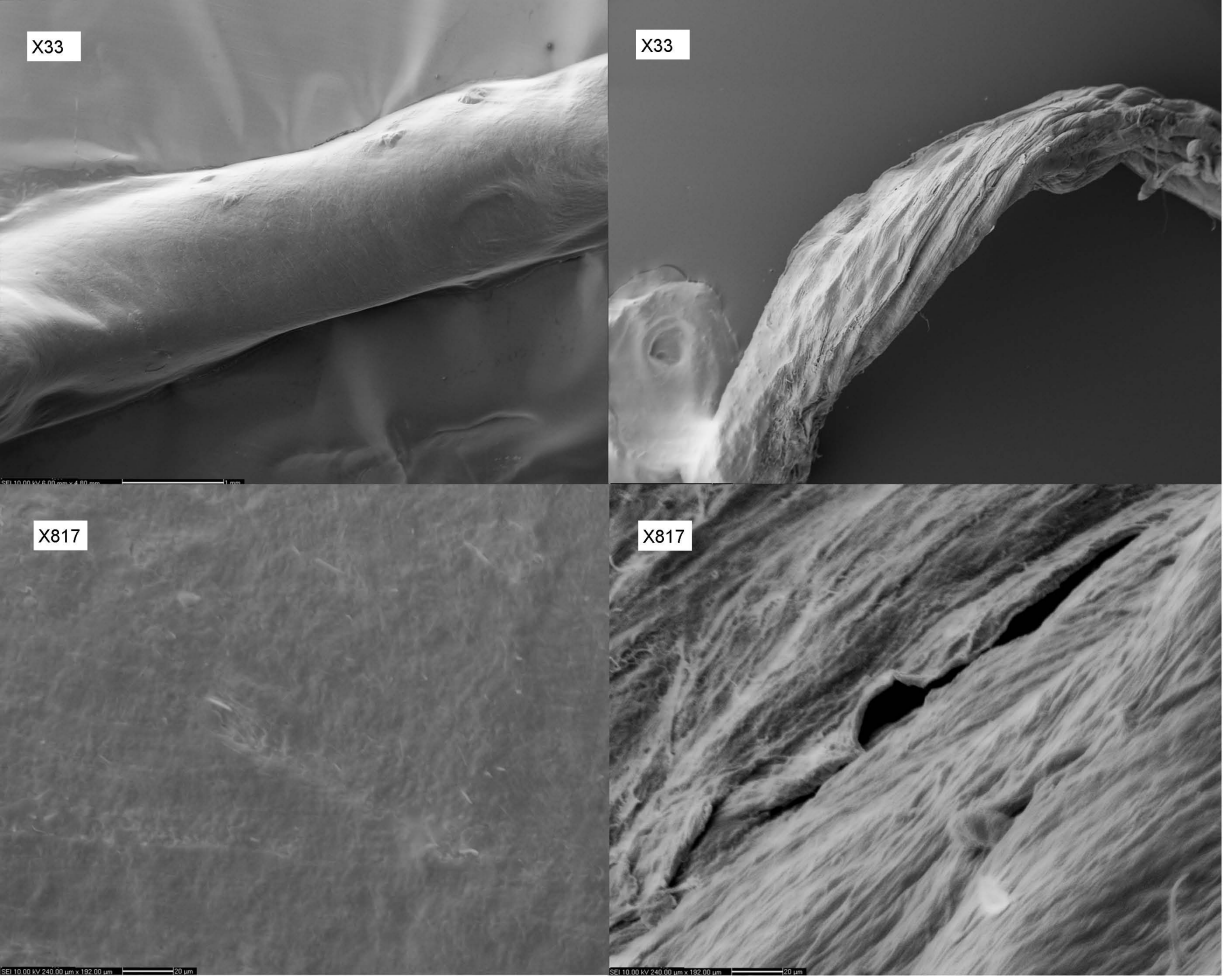
**Left: SEM of fresh blood vessel in a placenta (top × 33; bottom × 817)**. Right: SEM of formalin-fixed blood vessels of the placenta (top × 33; bottom × 817).

Initial MRA of formalin-fixed placentas showed that the MR contrast agents easily diffused to the interstitium (Figure 2). In contrast, MRA of fresh placentas showed that the contrast agent was confined to the blood vessels during the time of the experimental procedure. An increased permeability across the vessel endothelium was surprisingly found by scanning electron microscopy (SEM), where blood vessels from formalin-fixed and fresh placentas were investigated. SEM revealed large holes in the vessel walls of the formalin-fixed placentas, whereas the vessel walls of the fresh placentas were completely homogenous and dense (Figure 3), supporting the view that the reorganization of the vessel's microvasculature following protein binding to formalin leads to increased endothelial permeability. To our knowledge this observation is novel and could be an important consideration when performing angiography on formalin-fixed tissue in general.

### Human placenta

**Figure 4:**
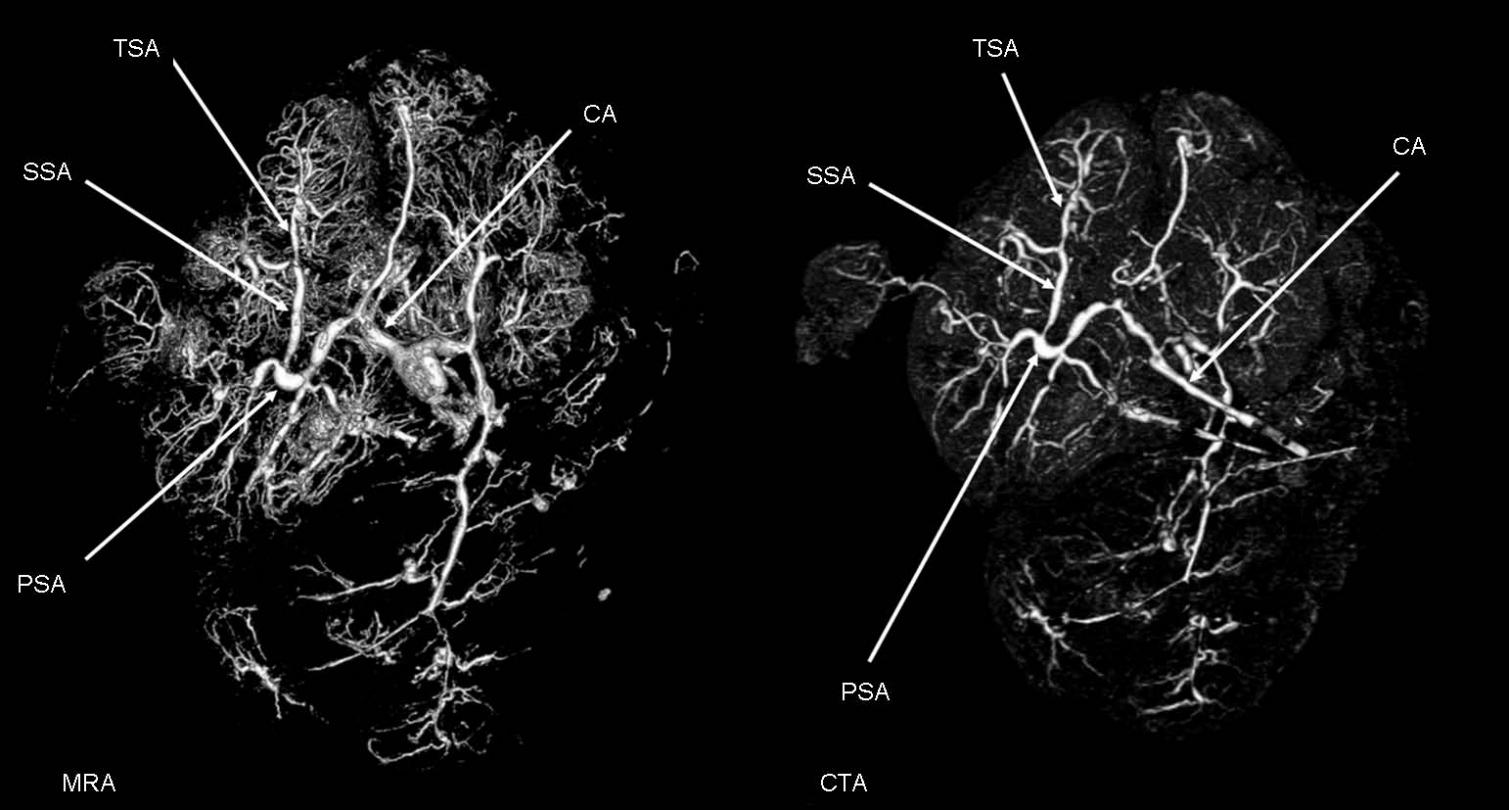
**Left: MRA of human placenta; 3D reconstruction made using Osirix software viewed from the foetal side of the placenta**. Key: CA, chorionic artery; PSA, primary stem artery; SSA, secondary stem artery; TSA, tertiary stem artery. Right: CTA of human placenta; 3D reconstruction made using Osirix software viewed from the foetal side of the placenta. Key: CA, chorionic artery; PSA, primary stem artery; SSA, secondary stem artery; TSA, tertiary stem artery.

A placenta was harvested immediately after elective caesarean section and placed in a water bath at 37°C. The umbilical cord was cut off approximately 5 cm proximal to the insertion to the placenta. The amnion was cut off as well. The blood vessels of the umbilical cord were catheterized using three venous catheters (size 17). The venous catheters were fixed to the blood vessels using polyester sutures (2-0 Ethicon) and the umbilical cord was clamped using a suture to prevent reflux of the injected solutions. The placenta was then perfused using saline with 5000 IU/L heparin [11]. The solution was heated to 37°C and 500 mL was injected into the two umbilical arteries with a pressure-controlled pump using the sphygmanometric principle. Contrast mix 1 was then injected using the pump with a pressure ≤ 60 mmHg [12]. During injection, the placenta was placed in a water bath at a temperature of 35-40°C to ensure that the contrast solution remained liquid. It was then positioned in the MR scanner and the 3D MRA protocol was followed by subsequent fixation with 4% formalin or 2.8% FineFix (Milestone Medical Technologies, USA). MRA showed vessels from a number of different generations starting with the cotelydonary arteries branching off into primary, secondary and tertiary stem vessels: even 6th generation blood vessels were visible. Subsequently, the placenta was subjected to CTA (Figure 4), which also showed branching of the vessels down to the 6th generation of stem vessels. In general, MRA was capable of showing considerably more very small vessels than CTA, giving the overall impression that the quality of MRA was as good or better than the results of conventional CTA.

### *Monopterus albus*

**Figure 5:**
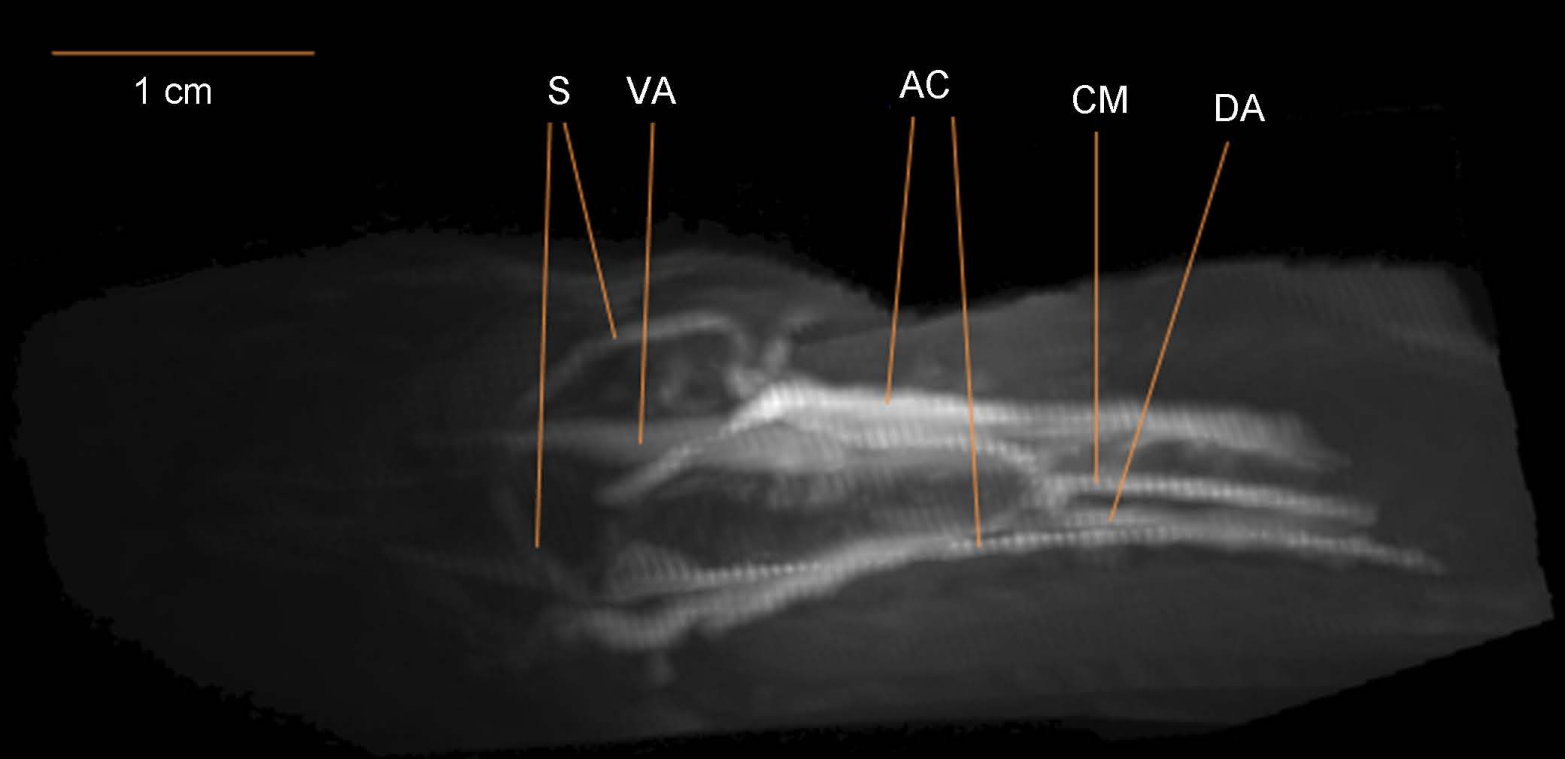
**Dorsal view of the region 3-6 cm caudal to the snout of *Monopterus albus *perfused with contrast mix 2, with anterior to the left**. The image was created by 3D-rendering software following MRI. Key: VA, ventral aorta; DA, dorsal aorta; S, shunt vessels joining ventral and dorsal aorta; CM, celiacomesenteric artery; AC, anterior cardinal veins.

**Figure 6:**
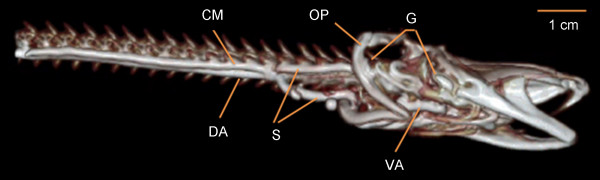
**CTA of *Monopterus albus *using Microfil contrast medium showing a lateral view of the head region; anterior to the right**. Image created by 3D rendering software following CT. Key: VA, ventral aorta; DA, dorsal aorta; S, shunt vessels joining ventral and dorsal aorta; CM, celiacomesenteric artery; G, right gill region; OP, right operculum.

Specimens of *Monopterus albus *(weight 150-200 g; length 50-55 cm) were anesthetized and euthanized with 1 g/L benzocaine. The pericardium and ventral aorta were exposed with a ventral cut 7-12 cm caudal to the snout of the animal. The ventral aorta was catheterized and saline with 5000 IU/L heparin was infused at a pressure of 30 mmHg [13] until all blood had been replaced. Intravascular perfusion was performed with either contrast mix 1, contrast mix 2 or Microfil. The body was cooled with ice to solidify the injected contrast agent. MRA was performed in animals injected with contrast mixes 1 or 2 (Figure 5) and CTA was performed in animals injected with Microfil (Figure 6). All MRA and CTA agents, particularly contrast mix 2, were able to delineate major vessels in the head, such as the ventral and dorsal aortae, the connected shunt vessels and the celiacomesenteric artery. Thanks to the low viscosity of contrast mix 2, it was possible to delineate the small capillary beds in the head, allowing a 3D representation of the anterior cardinal veins (Figure 6). The advantage of MRA over CTA was its ability to image the vessel structures without including the bone and cartilage, which is of particular importance in this species where the blood vessels and bones lie in close proximity.

### Porcine heart

**Figure 7:**
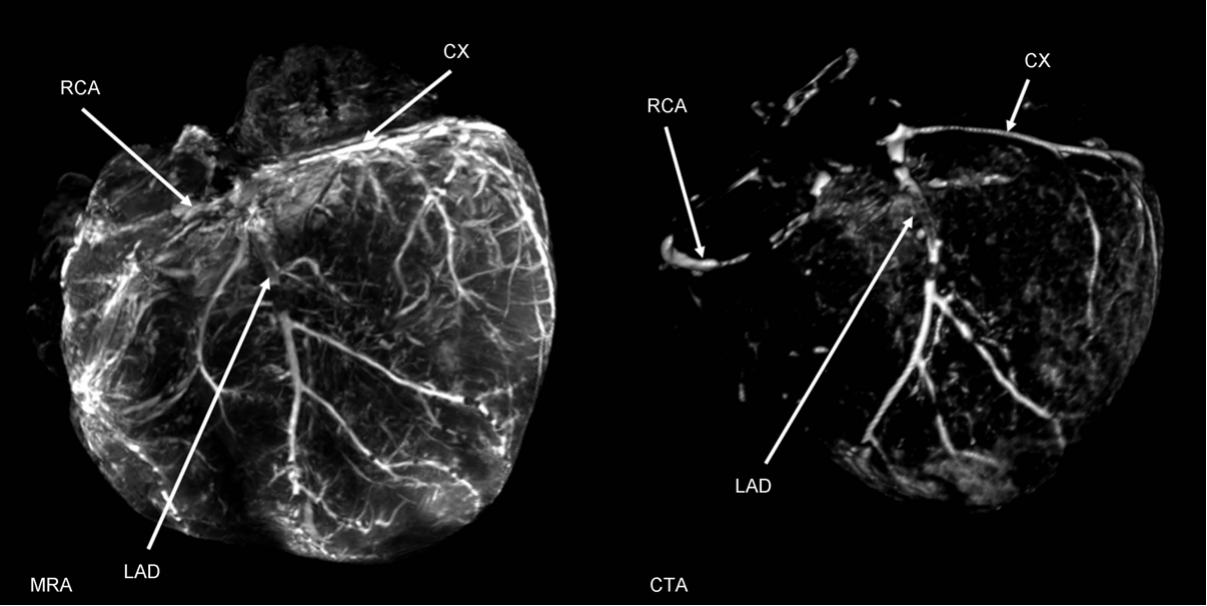
**MRA and CTA of the same porcine heart**. Left: MRA of heart from female Danish Landrace pig, visualized in a 3D view. Key: LAD, left anterior descending artery; CX, circumflex artery; RCA, right coronary artery. Right: CTA of the heart visualized in a 3D view. Key: LAD, left anterior descending artery; CX, circumflex artery; RCA, right coronary artery.

One female Danish Landrace pig weighing 65 kg was used for the experiment. Following anaesthesia the pig was moved to the surgical unit where 100 IU/kg heparin was administered through an ear vein cannula and allowed to circulate in the bloodstream for 5 min before euthanasia was induced by an overdose of phenobarbital. Subsequently, thoracotomy was performed and the heart was removed. The proximal part of the left anterior descending artery and right coronary artery were dissected free and a suture was placed around the arteries. A cannula (inner diameter 2.5 mm) was introduced into the proximal part of each artery and sutures were tightened around them to prevent retrograde flow of the contrast solution. Next, the coronary arteries were perfused with contrast mix 1, ex-vivo MRA was performed and the heart was perfused with 2.8% FineFix prior to CTA (Figure 7). MRA provided detailed information about the blood vessel anatomy and showed the smallest blood vessels in considerably greater number than CTA. Both MRA and CTA showed the right coronary artery (RCA), left anterior descendens (LAD) and the circumflex branch (CX).

### *Trachemys scripta*

**Figure 8:**
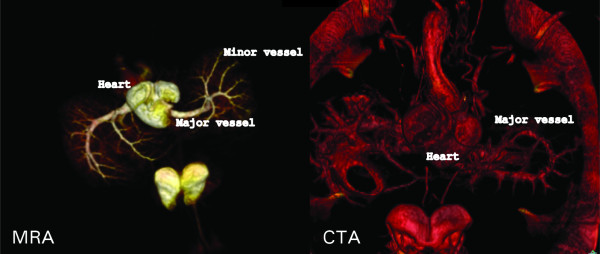
**MRA and CTA of a turtle**. Left: MRA in a 3D view. Right: CTA in a 3D view.

To test whether MRA is feasible for whole animals, two turtles (*Trachemys scripta*) were perfused with heparinized saline followed by perfusion with contrast mix 1. Perfusion was performed from both arterial and venous sides to make filling of the blood vessels more complete. Next, each animal was cooled with ice to solidify the injected contrast mix. MRA and CTA were performed. Figure 8 shows a 3D reconstruction of the images obtained with MRA and CTA, demonstrating that MRA provided several advantages over CTA. Smaller branches of the blood vessels could be observed by MRA, whereas the turtle shell absorbed the emitted X-ray radiation in CTA, making it difficult to depict blood vessels in close proximity to the shell.

## Conclusions

We have demonstrated a novel experimental method to generate a 3D representation of blood vessels using MRA specifically applied to ex-vivo studies. We found that MRA provided detailed information similar to or better than conventional CTA. MRA has a great advantage over CTA in its ability to visualize *only *the vessel configuration without interfering signals from adjacent bones as seen in CTA. In all cases, we found that the best method to obtain high-quality ex vivo MRA was to perfuse the blood vessels with saline containing heparin at 40°C immediately after organ harvesting, then lowered into a water-bath at 40°C and perfused with contrast solution. In cases where both MRA and CTA must be performed on the same specimen, contrast mix 1 is to be preferred whereas in cases where only MR scans are performed contrast mix 2 is preferable. It was observed that placental vascular tissue becomes leaky when formalin-fixed, leading to increased permeability and outward diffusion of MR contrast agent.

## Methods

### Ethics

This study complied with the Helsinki Declaration. Approval to use human placentas was given by the The Danish National Committee on Biomedical Research Ethics (approval #M-20080152), and all pregnant woman gave their written consent for the use of their placenta. Approval to include animals in this study was given by the The Danish Ministry of Justice for Animal Experiments Inspectorate (approval #2008-561-1522).

### MRA and CTA

MRA was performed with clinically available MR systems, a GE Horizon Echospeed LX 1.5 T (GE Healthcare, United Kingdom) and a Philips Achieva 1.5 T (Philips Medical Systems, Netherlands). Each specimen was positioned in a quadrature radiofrequency head coil. A fast localizer scan was followed by a high-resolution 3D gradient-echo sequence with the following parameters: field-of-view depending on specimen; thickness 1 mm; TR 23.1 ms; TE 1.6 ms and excitation flip angle 30°. A stack of multiple slices (with no gaps) was acquired, covering the entire specimen of interest with scan durations of 30-60 min. Image resolution was 0.5 × 0.5 × 0.5 mm^3^

CTA was performed using a 64-slice Siemens Somatom Definition (Siemens Medical Solutions, Germany) with dual source capacity. Acquisition parameters included a slice collimation of 4 mm; a pitch of 2°; 32 rotations (resulting in a 25.6 cm scanning volume) and a matrix size of 512 × 512. Acquisition parameters using the Somatom Volume Zoom option included a slice collimation of 4 × 1 mm, a rotation time of 0.5 s, 5-8 mm table feed/rotation, a matrix size of 512 × 512 and scan duration of 25-30 s. Transverse images were reconstructed with a section thickness of 1.25 mm and were reconstructed at 0.6 mm intervals.

Data acquired both by MRA and CTA were exported to DICOM format and a 3D reconstruction was performed using either the Mistar http://www.apollomit.com or the Osirix software http://www.osirix-viewer.com, allowing a maximum-intensity-projection in arbitrary directions.

### SEM

Two tissue samples were withdrawn from fresh placental blood vessels and two from formalin-fixated placenta blood vessels. The samples were freeze-dried and mounted on sample holders. The sample holders were placed in an Edward's gold sputter-coater, which covered the sample surface with a thin layer of gold. Subsequently, the samples were placed in a CamScan MaXim 2040 EnVac SEM (CamScan, United Kingdom) and images were obtained using secondary electrons and an Everhart-Thornley detector.

## Authors' contributions

MP constructed the MR sequence appropriate for MRA. HL performed MRA and CTA on the rice field eel. SFP performed porcine surgery. ASR performed MRA on the human placenta and the porcine heart. MP and TW performed MRA on the turtles. LU and LWTB performed CTA on the human placenta, turtle and porcine heart. BGJ, TW, NU, MP, HL, CL and ASR participated in designing the study. MP, HL and ASR participated in drafting the manuscript. All authors read and approved the final manuscript.
